# BORIS/CTCFL is an RNA-binding protein that associates with polysomes

**DOI:** 10.1186/1471-2121-14-52

**Published:** 2013-11-26

**Authors:** Babatunji W Ogunkolade, Tania A Jones, Johan Aarum, Jaroslaw Szary, Nicholas Owen, Diego Ottaviani, Muhammad A Mumin, Shyam Patel, Christopher A Pieri, Andrew R Silver, Denise Sheer

**Affiliations:** 1 Centre for Neuroscience and Trauma, Queen Mary University of London, Blizard Institute, Barts and the London School of Medicine and Dentistry, London, E1 2AT, UK; 2 Centre for Digestive Diseases, Queen Mary University of London, Blizard Institute, Barts and The London School of Medicine and Dentistry, 4 Newark Street, London, UK

**Keywords:** CTCF, Ribosomes, Nucleoli, WNT signalling

## Abstract

**Background:**

BORIS (CTCFL), a paralogue of the multifunctional and ubiquitously expressed transcription factor CTCF, is best known for its role in transcriptional regulation. In the nucleus, BORIS is particularly enriched in the nucleolus, a crucial compartment for ribosomal RNA and RNA metabolism. However, little is known about cytoplasmic BORIS, which represents the major pool of BORIS protein.

**Results:**

We show, firstly, that BORIS has a putative nuclear export signal in the C-terminal domain. Furthermore, BORIS associates with mRNA in both neural stem cells and young neurons. The majority of the BORIS-associated transcripts are different in the two cell types. Finally, by using polysome profiling we show that BORIS is associated with actively translating ribosomes.

**Conclusion:**

We have demonstrated the RNA binding properties of cellular BORIS and its association with actively translating ribosomes. We suggest that BORIS is involved in gene expression at both the transcriptional and post-transcriptional levels.

## Background

CTCF is a highly conserved and ubiquitous protein that has widespread functions in transcription regulation and chromatin architecture. It acts as a silencing and activating transcriptional factor, a chromatin insulator and a mediator of chromatin looping, and is essential for life [1,2]. Binding of CTCF to DNA is achieved primarily through its 11-zinc finger domain, which also facilitates protein-protein interactions [3-5]. CTCFL or BORIS (Brother of the Regulator of Imprinted Sites), is a paralogue of CTCF [1]. BORIS has almost identical 11 zinc finger domains to CTCF, and the proteins are thought to have evolved during vertebrate development from a gene duplication event [6]. However, the flanking N- and C-terminal regions of BORIS show no homology with CTCF or any other proteins [7]. BORIS also lacks the modular substrates for specific post-translational modifications that are critical for CTCF function, suggesting divergent roles for the two proteins. Indeed, *BORIS* and *CTCF* are expressed in a mutually exclusive manner during male germ-line development, suggesting that BORIS is involved in reprogramming the paternal DNA-methylation patterns [8]. Several lines of evidence suggest that BORIS plays a role in epigenetic regulation of gene expression. In tumour cell lines, where CTCF silences genes by DNA methylation, it has been shown that expression of BORIS can displace CTCF at these genes leading to local demethylation and gene activation [9-12]. Further epigenetic regulation is suggested by the binding of BORIS to the upstream binding factor (UBF), a transactivator of RNA polymerase I, which is involved in the maintenance of chromatin structure [13].

BORIS protein is readily detected in most cells and tissues [14], with abnormally high expression levels reported in several tumours and cell lines [15-22]. In contrast to previous findings suggesting divergence in the roles of BORIS and CTCF, recent evidence has shown that both proteins are able to mediate similar growth and tumour suppressor functions and both provide a protective effect during apoptosis [23]. This finding warrants further characterisation of the functional properties of BORIS.

We previously showed that BORIS is present both in the cytoplasm and nucleus, and is enriched in the nucleolus, a crucial compartment for ribosomal RNA and RNA metabolism [14]. The role of BORIS within the cytoplasm, which represents the major pool of BORIS protein in testis, has not been fully explored [24]. Here, we hypothesized that cytoplasmic BORIS interacts with RNA, as shown for certain other Zn-finger proteins [25,26], due to the subnuclear localisation of BORIS to the nucleolus, which is associated with RNA metabolism. To test this, we examined whether BORIS binds RNA and if so, whether this property changes in cells as they undergo phenotypic alterations. We show BORIS binds to distinct sets of RNA transcripts in neural stem cells and neurons and to a substantial amount of non-coding RNA. The transcripts are enriched for components of certain key cellular pathways including the WNT pathway. We further find that BORIS is associated with actively translating ribosomes. Together, our data suggest new roles for BORIS in the regulation of gene expression.

## Results

### BORIS is an RNA binding protein

**Figure 1:**
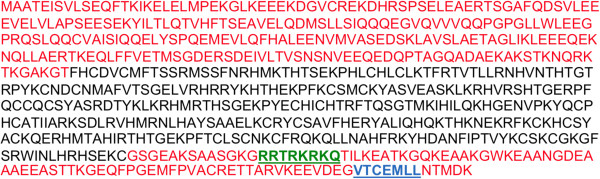
**Amino acid sequence of BORIS showing predicted nuclear localisation signal (green) and nuclear export signal (blue).** The unstructured N and C terminal regions [7] that flank the 11 Zinc-finger domains (black) are coloured red. In the C-terminal region, bold, underlined residues (green) represent a predicted nuclear localisation signal (NLS) (http://www.sbc.su.se/~maccallr/nucpred/cgi-bin/single.cgi). At the end of the C-terminal region, bold, underlined residues (blue) represent a predicted nuclear export signal (NES) (http://www.cbs.dtu.dk/services/NetNES/). The predicted export signal was identified when using the unstructured C-terminal (red) as input. No other NES were found in the other regions, i.e. the unstructured N-terminus or the Zinc-finger region.

Association of BORIS with newly synthesized RNA was first suggested by a run-on transcription assay on HEK293T cells, which showed that BORIS co-localises with 5-FU in punctate foci in both the nucleus and cytoplasm (Additional file 1: Figure S1). Analysis of the amino acid sequence of BORIS revealed the presence of a putative nuclear export signal (NES) in the C terminal region (Figure 1), indicating that the protein may shuttle between the nucleus and cytoplasm.

We therefore extended our investigation to determine whether BORIS interacts with RNA in other cell types and, if so, whether the interaction changes as cells undergo phenotypic alterations. We previously showed that BORIS is present at similar levels in hNP1 neural progenitor cells (Aruna Biomedical) and young neurons derived from hNP1 using well-defined culture conditions [14]. Gene expression arrays confirmed no significant change in expression of *BORIS* during neural differentiation (data available at NCBI’s Gene Expression Omnibus [27], accession number GSE42294). Expression of *BORIS* in hNP1 and HEK293T cells was confirmed by partial sequencing of PCR product (Additional file 2: Figure S2).

**Figure 2:**
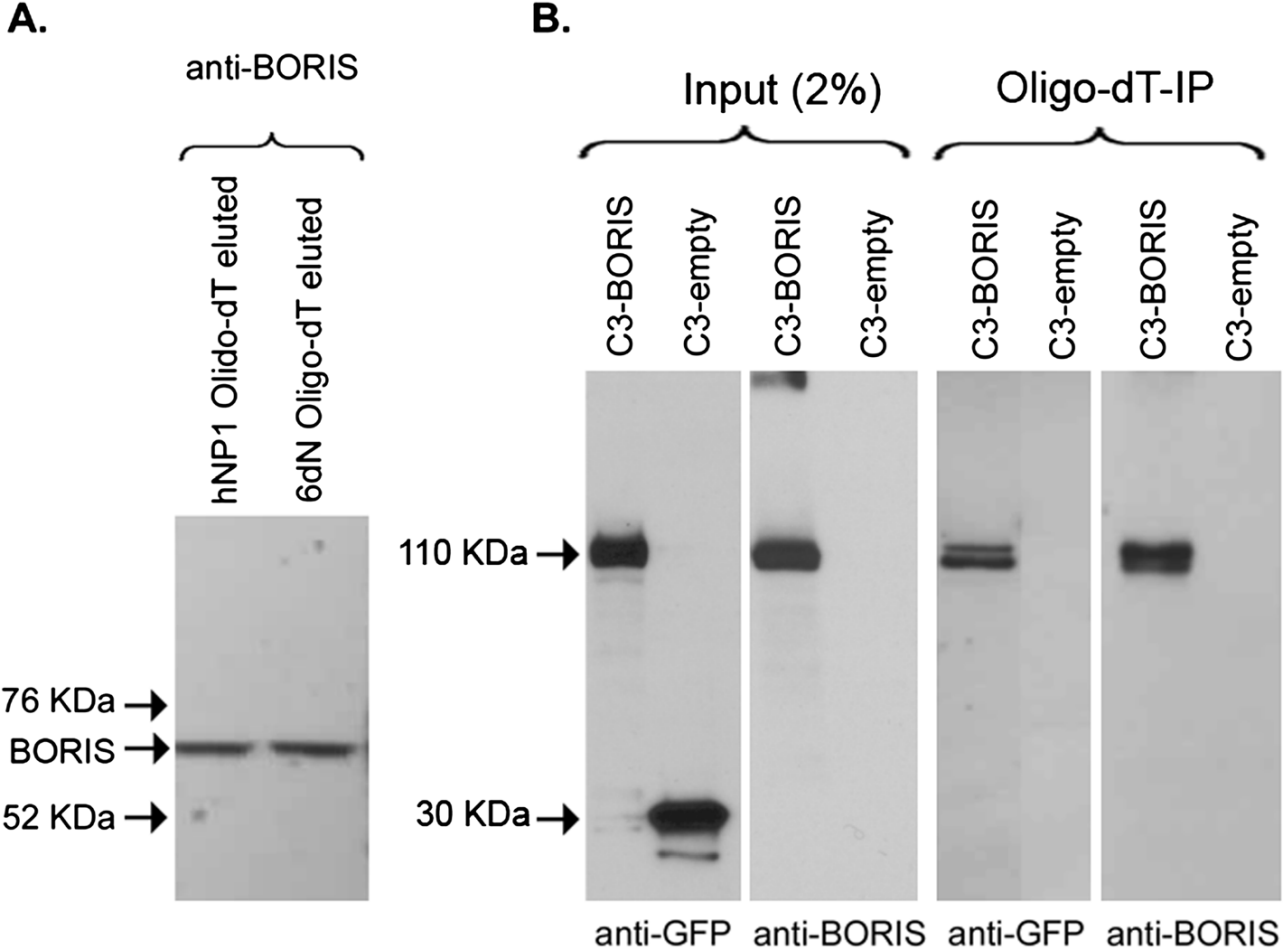
**Western blot analysis of oligo-dT-precipitated RNA-protein complexes. (A)** RNA-protein complexes form human neural progenitor cells (hNP1) and hNP1 cells differentiated to neurons over 6 days (6dN) were precipitated with oligo-dT beads analysed by SDS page western blot probed with anti-BORIS antibody (ab18337, Abcam). **(B)** Immuno-blotting of oligo-dT-RNA bound protein complexes from HEK293T cells transiently expressing BORIS (C3-BORIS) or empty vector (C3-empty). Western blot analysis of Input (2%) and oligo-dT bound protein complex probed with either anti- GFP or anti-BORIS.

To investigate if BORIS associates with endogenous RNA in hNP1 cells and hNP1 cells differentiated to neurons over 6 days (designated 6dN), we used oligo dT beads to precipitate mRNA from cell lysates and analysed co-precipitated proteins by Western Blot. In both cell types, BORIS was precipitated (Figure 2A), suggesting that the protein associates with mRNA. Similar results were obtained by oligo-dT-precipitation of protein complexes from HEK293T cells transiently expressing GFP-tagged BORIS protein, as detected by both anti- GFP antibodies and anti-BORIS antibodies (Figure 2B). No GFP was precipitated from cell lysates expressing GFP only (Figure 2B).

**Figure 3:**
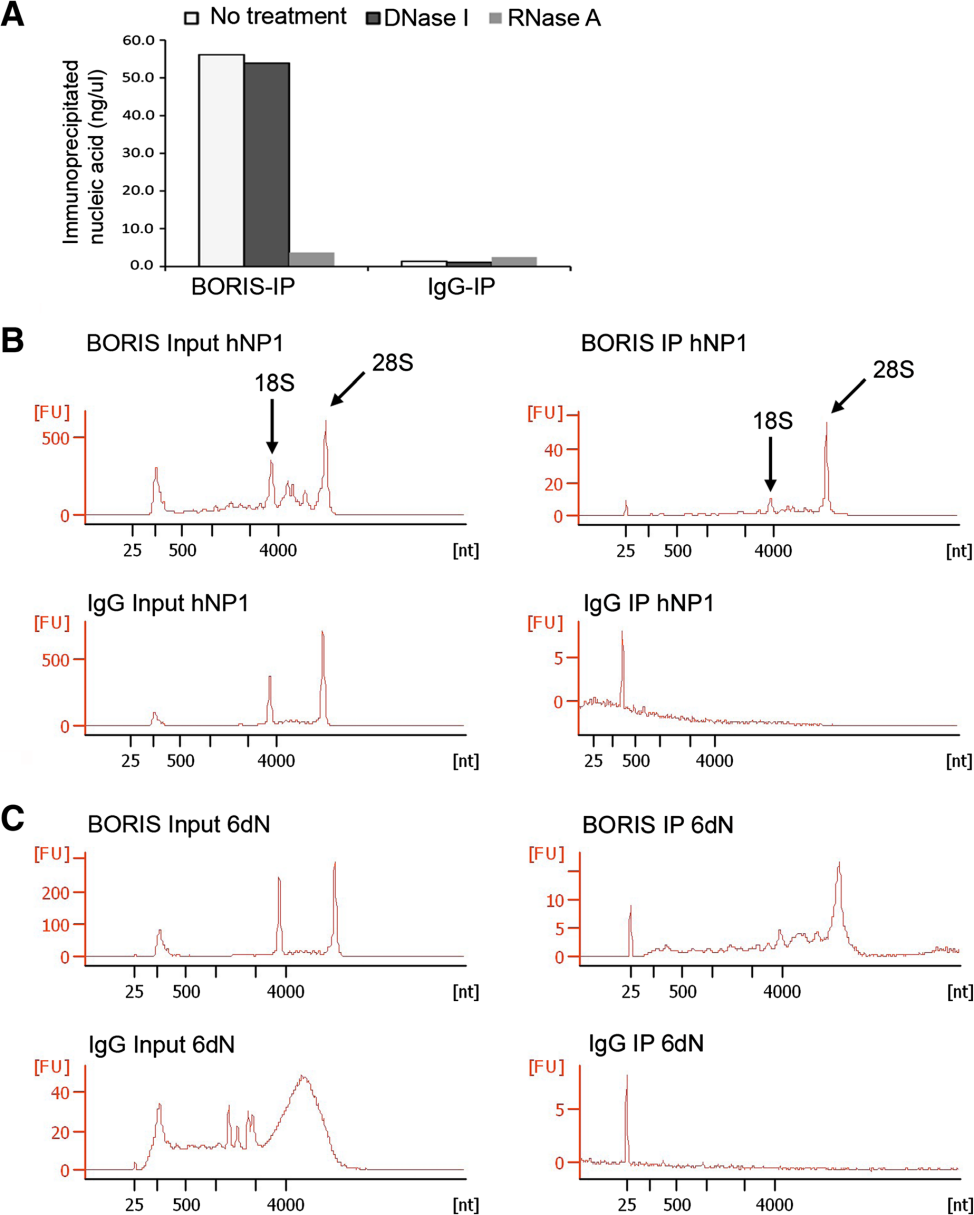
**Characterisation of immunoprecipitated nucleic acids. (A)** Quantification of BORIS and control IgG immunoprecipitated nucleic acids before and after digestion with either DNase I or RNase A. **(B and C)** Agilent Bioanalyser gel-electrophoresis of BORIS and control IgG precipitated RNA. The BORIS-precipitated samples revealed a prominent band migrating as 28S rRNA and a less prominent band as 18S in both hNP1 **(B)** and hNP1 cells differentiated to neurons over 6 days (6dN) **(C)**. Note the different scale of the Y-axis.

We then used native RNA-immunoprecipitation to isolate RNAs that were associated with BORIS. A substantial amount of nucleic acids (5-15% of input) was consistently immunoprecipitated from both hNP1 and 6dN cells. To verify that this was RNA and not contaminating DNA, since BORIS is known to bind DNA [28,29], we treated the immunoprecipitates with RNase A or DNase I and quantified the remaining nucleic acid. Only RNase A treatment decreased the amount of precipitated nucleic acids, while DNase I had no effect (Figure 3A). Gel electrophoresis analysis of BORIS-precipitated RNA revealed a prominent band migrating as 28S rRNA, and a weaker band as 18S, suggesting that BORIS associates with ribosomes (Figure 3B and C). In comparison, no detectable RNA was precipitated by non-specific IgGs (Figure 3B and C).

**Figure 4:**
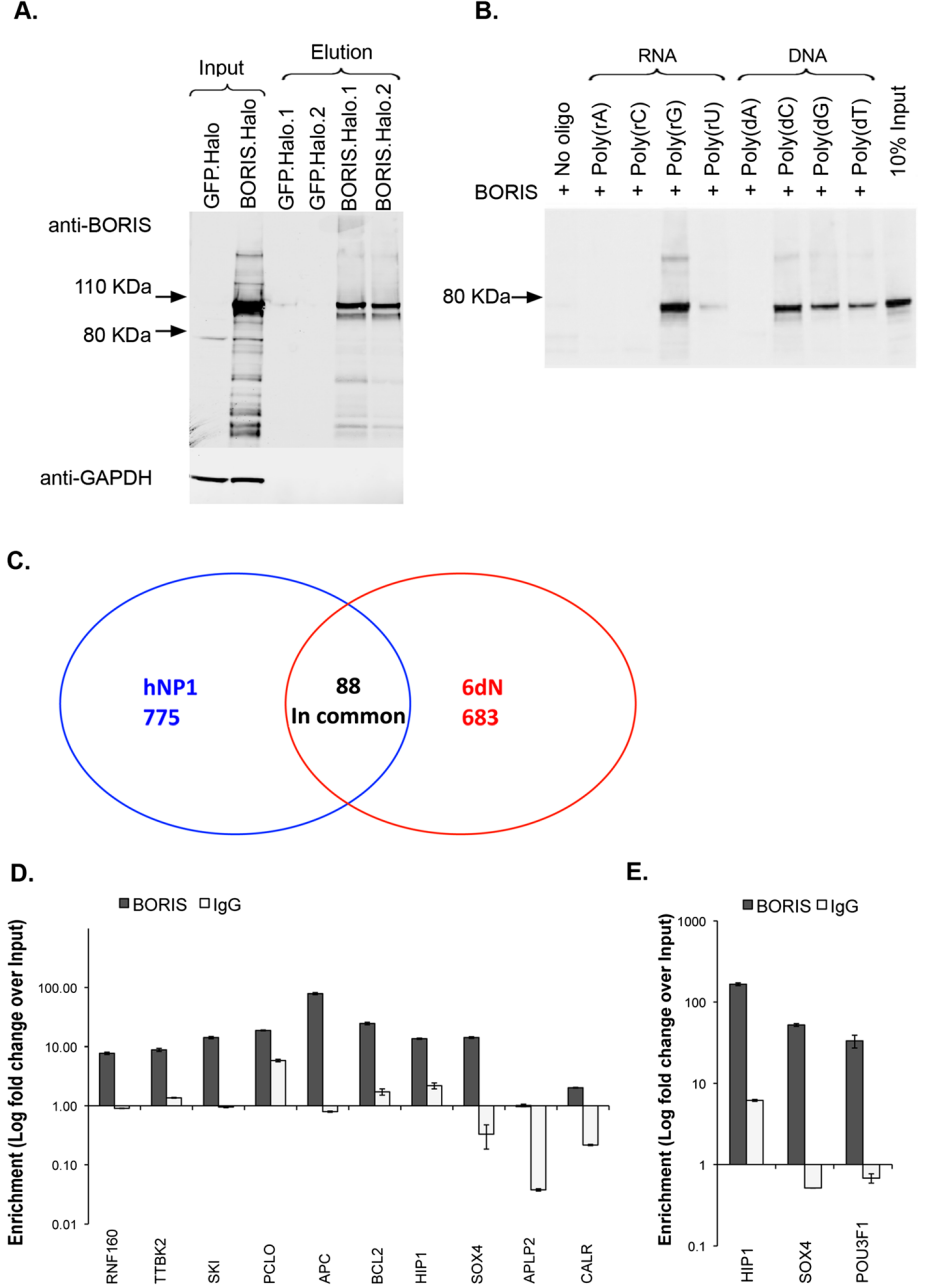
**BORIS binds directly to RNA in a nucleotide-specific manner. (A)** SDS-PAGE western blot analysis of the purification of Halo tagged BORIS from transfected HEK293T cells. **(B)***In vitro* binding assay using biotinylated RNA and DNA homopolymers and purified recombinant BORIS from **(A)**. BORIS showed association with poly(rG) and poly(rU), but not with poly(rA) or poly(rC). BORIS also showed specific affinity for poly(dT), poly(dG) and poly(dC) DNA while no binding was observed to poly(dA) or the streptavidin beads alone (No oligo, lane 1). **(C)** Venn diagram showing the number of identified BORIS associated transcripts in hNP1 and hNP1 cells differentiated to neurons over 6 days (6dN) as well as the number of transcripts associated with BORIS in both cell types. RT-qPCR confirmation of BORIS associated transcripts in hNP1 **(D)** and 6dN cells **(E)**. Data in **(D)** and **(E)** represent 2 technical replicates ± SD.

Next, to determine whether BORIS binds directly to RNA, a series of 20 mer RNA and DNA homopolymers with 3’ Biotin-TEG was utilised in an *in vitro* binding assay. Recombinant BORIS was purified from HEK293T cells (Figure 4A) and assayed for its ability to bind to the biotin-coupled homopolymers. As expected, we found that BORIS associates with the DNA homopolymers poly(dT), poly(dG) and poly(dC) [30]. In addition, BORIS also bound to poly(rG) and, to a lesser extent, to poly(rU) RNAs, while no binding was observed to polymers of rC or rA or to the streptavidin beads alone (Figure 4B). These experiments suggest that BORIS can interact directly with RNA.

### Identification of poly(A) RNAs bound to BORIS

To identify which transcripts were associated with BORIS in hNP1 and 6dN cells we immunoprecipitated the protein from cellular extracts. We then isolated the RNA and converted it to cDNA, which was hybridized to gene expression arrays. The signals from the arrays were then compared to those obtained from total RNA isolated from hNP1 and 6dN cells. Transcripts were scored as associated with BORIS if the fold change was larger than two and the *p*-value (ANOVA) was less than 0.01 (see Methods). In total, we identified 1097 and 962 probes representing 863 and 771 unique transcripts associated with BORIS in hNP1 and 6dN cells, respectively (Additional file 3: Table S1 and Additional file 4: Table S2). Of these, 88 transcripts were common to both hNP1 cells and 6dN cells (Figure 4C). These findings were confirmed for several genes by the validation of enrichment using RT-qPCR in hNP1 and 6dN cells (Figure 4D,E). In addition, we showed that the association of transcripts with BORIS did not correlate with their up- or down regulation during neural differentiation (Additional file 5: Table S3).

### Characterisation of BORIS-bound transcripts

**Figure 5:**
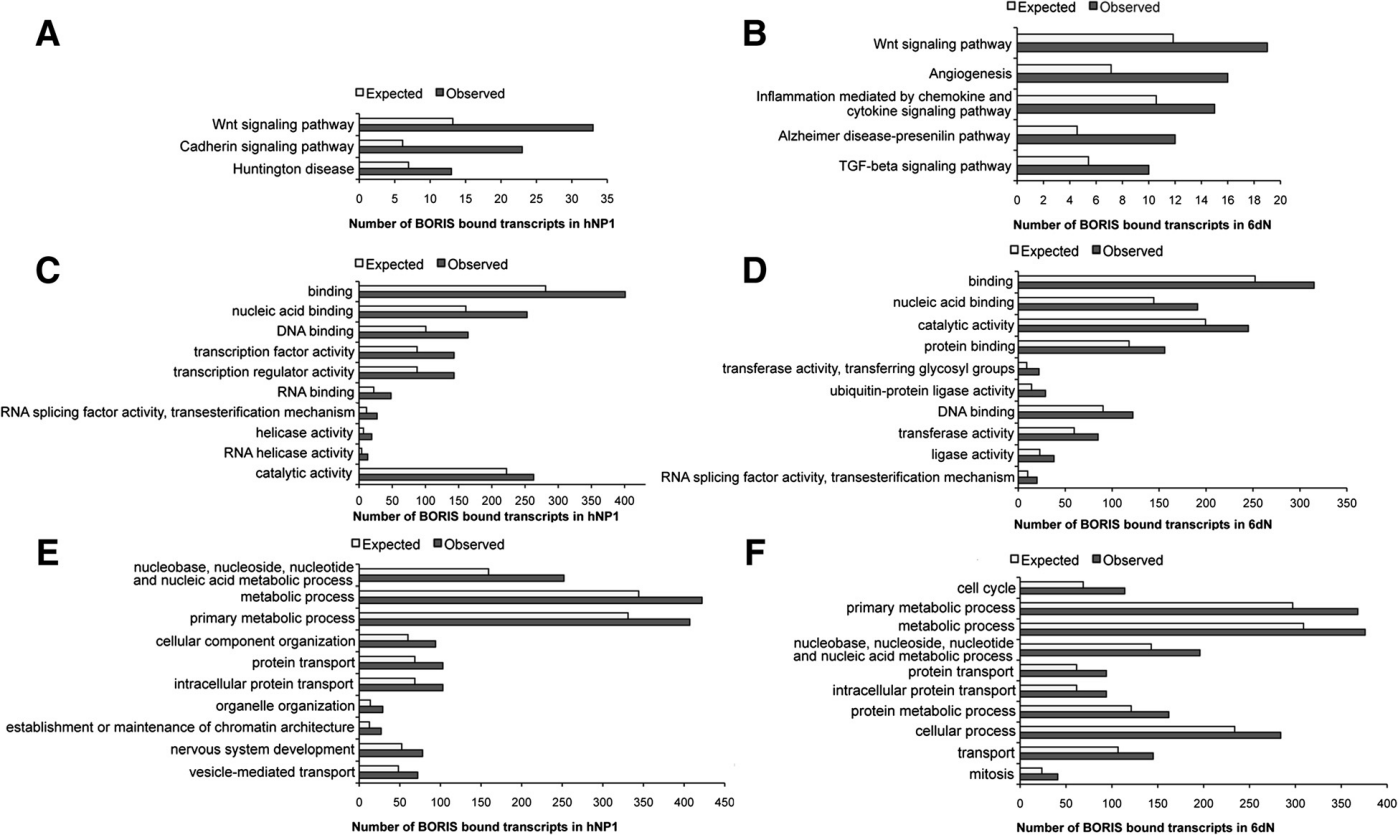
**Panther analysis of transcripts bound to BORIS.** Significantly enriched PANTHER annotations identified for BORIS-associated transcripts. Binomial distribution is applied to obtain *p*-values and *p*-values < 0.05 were considered. **(A,B)** Pathway analysis **(C,D)** Molecular function analysis and **(E,F)** Biological processes. Left panel hNP1 cells, right panel hNP1 cells differentiated to neurons over 6 days (6dN). Observed values indicate the actual number of transcripts form our lists assigned to a particular pathway/molecular function/biological process. The expected values indicate the number of genes expected from our lists for each PANTHER category, based on the human genome reference list.

We first used the PANTHER Protein Class Ontology platform [31] to identify over-represented pathways in each cell type. In hNP1 cells, significant enrichment (*p <* 0.05) was found for transcripts involved in *WNT signalling*, *cadherin signalling* and *Huntington disease* (Figure 5A and Table 1). In 6dN cells, significant enrichment (*p <* 0.05) was found for transcripts involved in *WNT signalling* as well as *angiogenesis*, *inflammation mediated by chemokines and cytokine signalling*, *Alzheimer disease-presenilin* and *TGF-β signalling* (Figure 5B and Table 1).

**Table 1:** ***p-*** **values for PANTHER analysis of pathways, molecular function and biological processes of transcripts bound in (A) hNP1 and (B) hNP1 cells differentiated to neurons over 6 days (6dN)**

**A.**		
	**BORIS bound transcripts in hNP1**	***p-*** **value**
**Pathway**		
WNT signaling pathway	33	0.00000258
Cadherin signaling pathway	23	0.00000012
Huntington disease	13	0.0252
**Molecular function**		
binding	401	6.54E-18
nucleic acid binding	253	1.49E-14
DNA binding	164	2.39E-10
transcription factor activity	143	3.48E-09
transcription regulator activity	143	3.48E-09
RNA binding	48	7.86E-07
RNA splicing factor activity, transesterification mechanism	27	3.56E-05
helicase activity	19	5.85E-05
RNA helicase activity	13	1.73E-04
catalytic activity	263	9.25E-04
**Biological process**		
nucleobase, nucleoside, nucleotide and nucleic acid metabolic process	252	8.72E-15
metabolic process	422	3.19E-08
primary metabolic process	407	5.66E-08
cellular component organization	94	1.45E-05
protein transport	103	2.76E-05
intracellular protein transport	103	2.76E-05
organelle organization	29	1.74E-04
establishment or maintenance of chromatin architecture	27	2.42E-04
nervous system development	78	3.60E-04
vesicle-mediated transport	72	5.89E-04
**B.**		
	**BORIS bound transcripts in 6dN**	***p*-value**
**Pathway**		
WNT signaling pathway	19	0.0325
Angiogenesis	16	0.00275
Inflammation mediated by chemokine and cytokine signaling pathway	15	0.115
Alzheimer disease-presenilin pathway	12	0.00257
TGF-beta signaling pathway	10	0.049
**Molecular function**		
binding	315	1.05E-06
nucleic acid binding	191	1.76E-05
catalytic activity	245	1.26E-04
protein binding	156	1.39E-04
transferase activity, transferring glycosyl groups	22	1.67E-04
ubiquitin-protein ligase activity	29	2.83E-04
DNA binding	122	3.80E-04
transferase activity	85	6.67E-04
ligase activity	38	2.03E-03
RNA splicing factor activity, transesterification mechanism	20	3.28E-03
**Biological process**		
cell cycle	114	7.90E-08
primary metabolic process	368	8.83E-08
metabolic process	376	4.35E-07
nucleobase, nucleoside, nucleotide and nucleic acid metabolic process	196	1.26E-06
protein transport	94	3.18E-05
intracellular protein transport	94	3.18E-05
protein metabolic process	162	5.45E-05
cellular process	284	5.66E-05
transport	145	7.51E-05
mitosis	41	6.49E-04

PANTHER [31] was then used for functional analysis of translated protein products for BORIS-associated transcripts. Significant enrichment (*p <* 0.05) was found in *DNA and RNA-binding proteins*, as well as *RNA splicing factor activity* in both hNP1 and 6dN cells (Figure 5C and D and Table 1). PANTHER analysis also showed that BORIS-associated transcripts are involved in diverse biological processes (Figure 5E and F and Table 1). Over-represented biological processes for transcripts from hNP1 include *metabolic process, cellular component organization, protein transport, organelle organization,* and *nervous system development.* Over-represented biological processes for transcripts from 6dN include *cell cycle, primary metabolic process, cellular process, transport (protein, ion etc.)* and *mitosis*.

### BORIS expression activates the β-catenin dependent WNT canonical pathway

**Figure 6:**
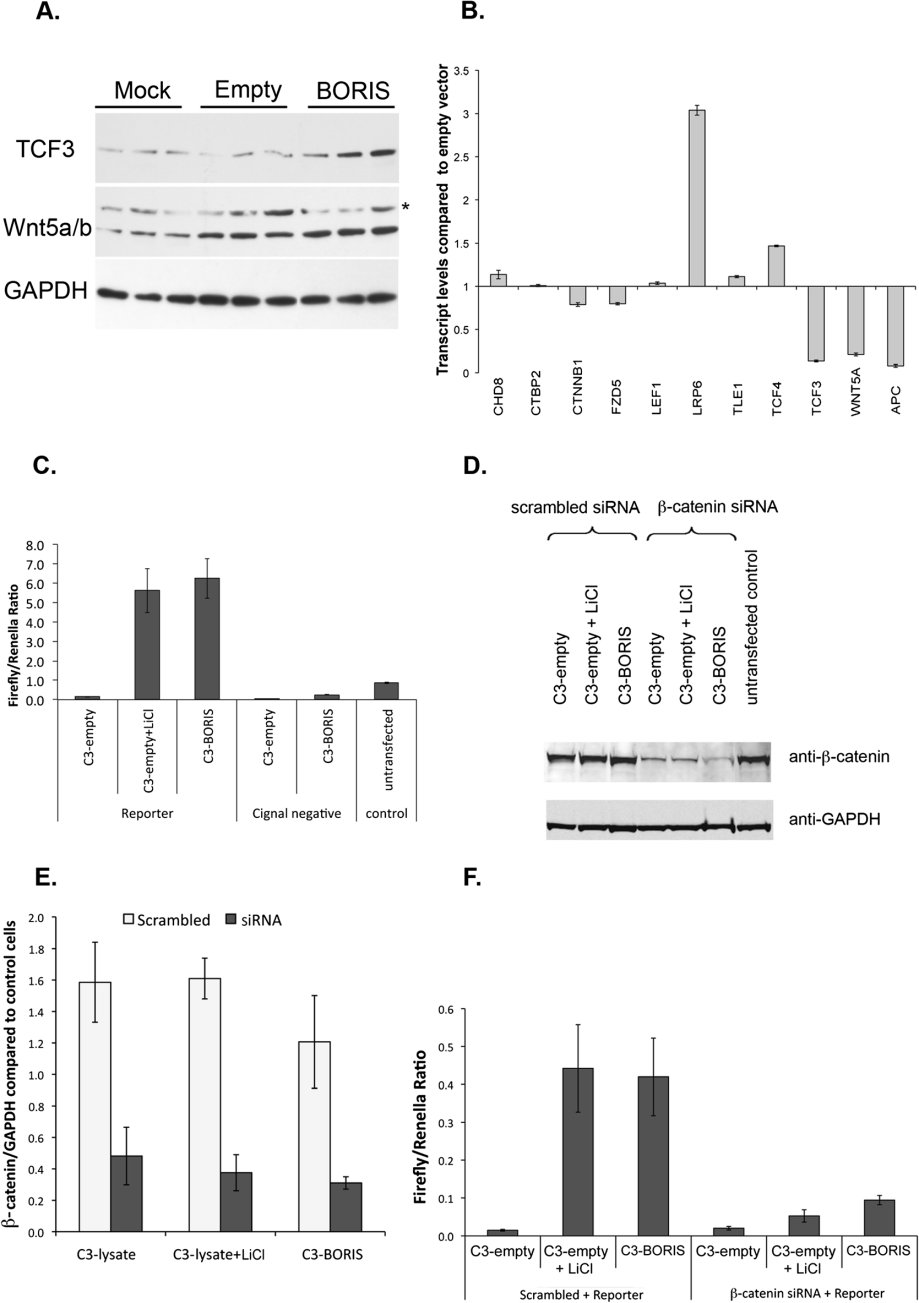
**Activation of the WNT pathway after BORIS over-expression. (A)** Western blot analysis of WNT5A/B and TCF3 in untransfected control, mock transfected (C3-empty) or BORIS transfected (C3-BORIS) HEK293T cells. The upper band in the WNT5A/B blot is non-specific (*) as shown by provider (Cell Signalling). The membranes were probed with the antibodies as indicated and GAPDH was used as a loading control. Lysates from three independent experiments were loaded on the same gel. **(B)** mRNA levels of BORIS bound transcripts in HEK293T cells over-expressing BORIS (C3-BORIS) as compared to empty vector (C3-empty). Data in **(B)** represent 2 technical replicates ± SD. **(C)** Luciferase activity of a TCF/LEF-responsive element reporter after BORIS (C3-BORIS) or mock transfection of HEK293T cells. Over-expression of BORIS (C3-BORIS) led to a more than four-fold increase in luciferase activity compared to cells transfected with empty vector alone (C3-empty). Cignal negative is a non-inducible reporter, lacking the TCF/LEF response element. LiCl was used as a general activator of the TCF/LEF reporter. Data are expressed as a mean ratio of the Firefly/Renilla luciferase activity (see Materials and Methods) and represents the average ± SD of 4 biological replicates. **(D)** Western blot analysis of HEK293T cells after over-expression of BORIS together with siRNA knock down of β-catenin. LiCl treated cells (C3-empty + LiCl) were included in the analysis to exclude a direct effect of LiCl on the expression of β-catenin. GAPDH was used as a loading control. **(E)** Densitometry quantification of β-catenin expression in **(D)**. Data represents the mean ± SD of 3 biological replicates. **(F)** Normalised TCF/LEF luciferase reporter activity after β-catenin knock-down and BORIS over-expression in HEK293T cells. β-catenin knock-down efficiently prevented BORIS-mediated activation of the TCF/LEF luciferase reporter, compared to Scrambled + Reporter with β-catenin siRNA + Reporter. Data shown is from one representative experiment.

In both hNP1 and 6dN cells, BORIS associates with several transcripts of the WNT pathway, including APC, TCF, lpd5/6, WNT5A and FZD5/10 (frizzled family receptor 5/10) (Additional file 6: Figure S4). To investigate if BORIS can influence this key pathway, we over-expressed BORIS in HEK293T cells and assessed the protein levels of a set of WNT pathway components. Over-expression of BORIS caused a significant increase in the amount of TCF3 (*p* < 0.05) and WNT5A/B (*p* < 0.01) protein (Figure 6A and Additional file 7: Figure S5). Whilst we observed a slight increase in nuclear β-catenin, this was not statistically significant and there was no overall increase in total cellular β-catenin protein following BORIS over-expression (Figure 6E). No change in protein levels was found for LEF1 and TCF4 WNT pathway components. Analysis of mRNA levels after BORIS over-expression showed no alteration for most WNT pathway components, while there was a significant decrease in expression for TCF3 (*p* < 0.02), APC (*p* < 0.007) and WNT5A (*p* < 0.03) (Figure 6B).

To determine directly if BORIS influences the activation of the WNT pathway, we then used a luciferase reporter assay where the luciferase expression is driven by tandem repeats of multiple copies of the consensus TCF/LEF- β-catenin responsive element (SABiosciences). LiCl, an inhibitor of GSK-3, was used as a positive control for pathway activation [32]. Transient over-expression of BORIS in HEK293T cells led to a more than four-fold increase in luciferase activity compared to cells transfected with empty vector alone (Figure 6C). This activation was dependent on β-catenin as siRNA knock-down of β-catenin caused a significant reduction in the effect of BORIS over-expression in the TCF/LEF luciferase assay (Figure 6D, E and F).

### BORIS associates with polysomes

**Figure 7:**
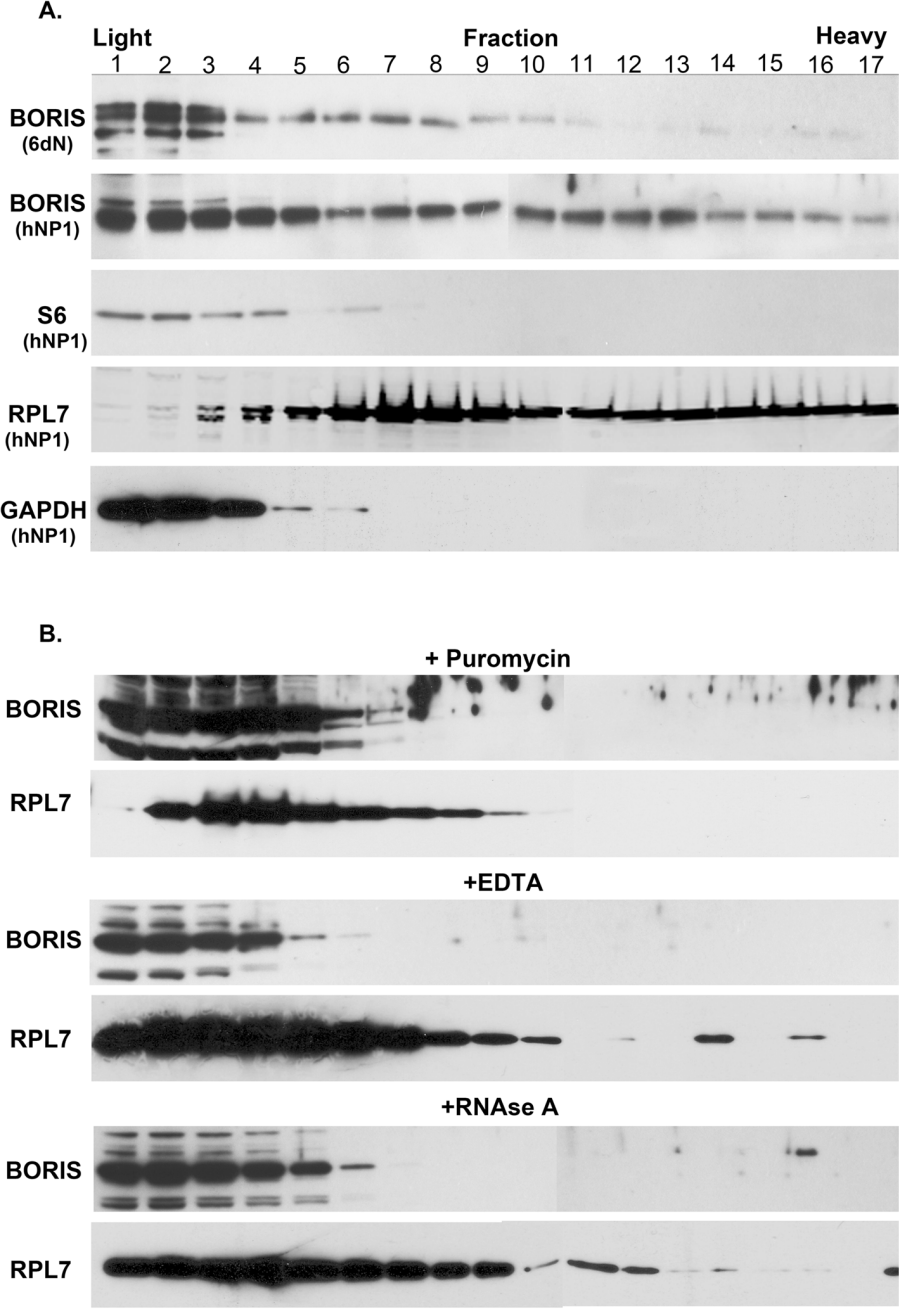
**Polysome profiling of BORIS. (A)** Polysome profiles of cell lysates from hNP1 and hNP1 cells differentiated to neurons over 6 days (6dN) on a 10-50% sucrose gradient. Lighter particles at the top of the gradient are shown on the left and heavier fractions are shown on the right. Equal volumes of each fraction were analysed by SDS-PAGE and probed with indicated antibodies. S6 and RPL7 are ribosome-associated proteins while GAPDH is not. **(B)** Polysome profiling of HEK293T cell lysates, detecting endogenous BORIS, after disruption of polysomes by puromycin (top panel), 30 mM EDTA (middle panel) or following RNAse A digestion (lower panel).

The large amount of RNA including ribosomal RNA, bound to BORIS, suggested that BORIS interacts with the translational machinery. To investigate this directly, we performed polysome profiling on cell extracts prepared from hNP1 and 6dN cells and analysed the distribution of BORIS in the resulting gradients by Western blotting. Consistent with a ribosomal association, BORIS was present throughout the gradient, co-sedimenting with all ribosomal subunits (40S and 60S) as well as monosomes (80S) and polysomes (Figure 7A). A similar sedimentation profile was observed for the ribosomal protein L7 (RPL7). The majority of BORIS was detected in the light fractions at the top of the gradient, where it co-sediments with the ribosomal proteins S6. The cytoplasmic but non-ribosome associated protein, GAPDH, was only detected in the light fractions.

Polysome profiling of HEK293T cells showed a similar sedimentation profile of BORIS to that observed in hNP1 and 6dN cells (Figure 7B). Inhibition of translation in HEK293T cells using puromycin, which causes premature chain termination and polysomal dissociation [33] shifted BORIS and RPL7 to the first, light fractions (Figure 7B). Furthermore, both RNase A digestion and dissociation of ribosomes into subunits by 30 mM EDTA with the concomitant release of mRNA and the 5S ribosomal protein [34,35], also shifted the sedimentation of BORIS and to a lesser extent RPL7 towards lighter fractions (Figure 7B). Together, these findings suggest that BORIS associates with actively translating ribosomes in these cells.

## Discussion

Here, we provide evidence that BORIS, best known for its role in DNA binding and transcriptional regulation, also binds RNA *in vitro* and associates with subsets of mRNAs and with translating ribosomes in neural stem cells and young neurons. The ability to bind to both DNA and RNA is not unique to BORIS, and is a feature of certain other zinc finger containing proteins [36-41]. The zinc finger domains of BORIS, with which it associates with DNA, are almost identical to those in CTCF and the proteins are reported to share DNA binding sites in the genome [8,11,24]. A recent study has suggested that the zinc fingers in BORIS are needed for both nuclear and nucleolar localisation [42]. It remains to be established whether the zinc finger motifs are important for the RNA-binding properties of BORIS, as is the case for TFIIIA [37], WT1 [43] and certain other proteins [44].

An interesting feature of BORIS is that its mRNA expression is extremely low in cultured or primary cells, yet the protein levels are readily detectable. This is consistent, however, with a report that RNA binding proteins tend to exhibit high protein stability and translational efficiency, yet their transcripts have a short half-life [45]. The authors of the report suggest that tight regulation of the levels of RNA binding proteins is required since a significant change in their expression may affect many targets altering global expression levels.

Although the majority of BORIS-associated transcripts differ between hNP1 and 6dN cells, similarities are observed in the pathways in which the transcripts are involved in the two cell types. For example, BORIS-associated transcripts in both cell types encode proteins involved in the canonical WNT pathway. WNT signalling is crucial in the regulation of a wide range of cellular processes such as apoptosis, cell proliferation, and differentiation, including that of neural stem cells [46-48].

A role for BORIS in regulating WNT signalling is supported by our finding that BORIS increases the activity of a TCF/LEF reporter following transient over-expression in HEK293T cells (Figure 6C). As the reporter activation is dependent on β-catenin (Figure 6F), BORIS is unlikely to affect the TCF/LEF reporter directly, but rather to have a post-transcriptional role. BORIS associates with several transcripts coding for regulatory components of the pathway and it is therefore conceivable that its over-expression may affect the translation of WNT pathway components. Indeed, BORIS over-expression leads to increased TCF3 and WNT5A protein levels, whilst their respective transcript levels are decreased (Figure 6A and B) Although there are several possible explanations for this increase in protein levels, for example post-translational modifications leading to greater protein stability [49,50], the fact that BORIS associates to these transcripts as well as to actively translating ribosomes (Figure 7A,B) argues for a translational effect of BORIS on these proteins. However, further studies are required to conclusively answer this question.

The biological consequences of the association of BORIS with different transcripts within individual pathways in hNP1 and 6dN cells have yet to be determined. BORIS may be involved in coordinated regulation of different transcripts within certain pathways at specific time points of cell development or differentiation.

## Conclusion

We show that BORIS can directly interact with RNA *in vitro* and is associated with a subset of mRNA and translating ribosomes in neural stem cells and young neurons. Transient over-expression of BORIS increases the protein levels of several BORIS-associated transcripts without any concomitant increase in transcript levels suggesting a role for BORIS in translational control.

## Methods

### Cell culture

Human neural stem cells, hNP1, derived from the cell line WA09 (46,XX) (Aruna Biomedical, hNP7013.1), were cultured in Neurobasal medium (Invitrogen, 21103–049) supplemented with B27 (Invitrogen, 12587–010), FGF-2 10 ng/ml, 1% penicillin/streptomycin and 2 mM glutamine as previously reported [14]. Half the medium was changed every other day. We induced differentiation by omitting FGF-2 from the medium as described by Shin et al., [51]. Human embryonic kidney cells, HEK293T, were maintained in RPMI containing 10% fetal bovine serum 1% penicillin/streptomycin (Invitrogen) and 2 mM glutamine at 37°C in 5% CO_2_ (Invitrogen).

### Antibodies

BORIS antibody ab18337 (1:1000 dilution, Abcam), CTCF antibody 07–729 (1:1000 dilution, Millipore) and GAPDH antibody 14C10 (1:1000 dilution, Cell Signaling) were used in Western data shown. The specificity of the BORIS antibody was determined using recognition of GFP-tagged recombinant BORIS and non-recognition of GFP-tagged recombinant CTCF protein by western blotting (Additional file 8: Figure S3). The specificity of the BORIS antibody has also previously been confirmed by siRNA knock down, peptide competition and the recognition of recombinant BORIS [14]. WNT3a (C64F2) rabbit monoclonal antibody, WNT5a/b (C27E8) rabbit monoclonal antibody and LRP6 (C5C7) rabbit monoclonal antibody were from the WNT signaling antibody sampler kit, 2915 (Cell Signaling) and TCF3 (D15G11) rabbit monoclonal antibody and TCF4 (C48H11) rabbit monoclonal antibody were from the TCF/LEF1 antibody sampler kit, 9383 (Cell Signaling) and were used at 1:1000 dilution.

### Run-on transcription assay

For immunodetection of newly synthesized RNA, HEK293T cells grown on coverslips were briefly incubated (15–20 minutes at 37°C) with 2 mM 5'-fluorouridine (5-FU) (F5130; Sigma) [52]. Cells were then fixed with 4% paraformaldehyde for 10 min, permeabilised with 1% Triton X-100, and incorporation of 5-FU into nascent RNA was monitored using antibody against halogenated UTP (1:100, anti-bromodeoxyuridine (BrdU) clone BU-33, B8434; Sigma) and a Texas Red-conjugated secondary antibody. Nuclei were stained with 0.1 mg/ml 4’, 6-Diamidino-2-phenylindole (DAPI, Molecular Probes) and mounted in Mowiol (Calbiochem). For standard 2 dimensional analysis, specimens were visualized using a Zeiss Axiophot microscope equipped for epifluorescence using Zeiss plan-neofluar 100x objective. Separate grey-scale images were recorded with a cooled CCD-camera (Hamamatsu). Image analysis was performed using SmartCapture X software (Digital Scientific).

### Identification of nuclear export signal

Identification of a putative nuclear export signal (NES) in the C terminal region was performed using NetNES (http://www.cbs.dtu.dk/services/NetNES/).

### Oligo-dT precipitation of BORIS

Cells were trypsinised, washed in ice cold buffer A (10 mM Tris–HCl pH 7.5, 1.5 mM MgCl_2_, 0.2 mM EDTA, 10 mM KCl) and lysed in buffer C (10 mM Tris–HCl (pH 7.5), 100 mM NaCl, 2.5 mM MgCl2, 0.5% Triton X-100, and 2unit/μl RNaseOUT). 1000 μg of protein lysate was incubated with 100 μl oligo-dT-dynabeads (Invitrogen) and incubated at 4°C for 30 minutes. Oligo-dT/mMRNA/protein complex was separated from unbound proteins using an Invitrogen magnetic separator. The beads were washed five times with solution D (20 mM Tris–HCl pH 8.0, 150 mM NaCl, 0.02% NP-40 and 1U/ml RNaseOUT) using at least twice the lysate volume for washing. Beads and attached complexes were re-suspended in 20–40 μl PAGE loading buffer for western blot analysis.

### Identification of BORIS bound mRNAs

Immunoprecipitation of BORIS-mRNA complexes was used to assess the association of BORIS with target mRNAs as previously described with some modification [53]. Briefly, 10–20 million cells were washed with PBS and lysed in ice cold swelling buffer A (25 mM HEPES, 1.5 mM MgCl_2_, 85 mM KCl, pH 8.0) for 5 minutes. After spinning for 5 minutes at 4°C, the pellet was lysed in buffer C (buffer A supplemented with 0.2% NP-40, 1% Triton X-100, with 0.01% saponin, 1 × protease inhibitor cocktail (Roche), 2 U/ml of RNaseOUT and phosphatase inhibitors mix (5 mM each of Sodium Fluoride, Sodium Orthovanadate and beta-glycerophosphate)) for 30 minutes and cleared by centrifugation at 21,000 g for 10 minutes. The cleared supernatant was incubated with 10 μg BORIS antibody (ab18337, Abcam) coupled to dynabead protein A (100 μl) (Invitrogen) for 1–2 hours at 4°C. After extensive washes with buffer D (buffer A, supplemented with phosphatase inhibitors mix, 1 × protease inhibitor cocktail (Roche), 0.1 U/ml of RNaseOut, 0.02% NP-40 and 0.25% Triton X-100), the bead-protein complex was incubated with 50 units of DNase 1 containing 100 units of RNaseOUT for 5 minutes at 37°C. An equal volume of proteinase K containing buffer was added and incubated for another 15 minutes at 37°C. RNA was extracted with standard phenol chloroform procedure and precipitated with 2 μl of glycogen (Sigma-Aldrich).

The RNA was used for either hybridization to Affymetrix U133 plus 2.0 expression arrays or for RT-qPCR verification of BORIS target transcripts. For array analysis, double stranded cDNA was synthesized from 1.5 – 5 μg total RNA using the Affymetrix One-cycle cDNA synthesis kit following the manufacturer’s instructions (Affymetrix). Synthesis of Biotin-labeled cRNA was performed using the Affymetrix GeneChip IVT labeling kit followed by purification with the sample cleanup module. Labeled cRNA was then fragmented and hybridized to Affymetrix GeneChip Human Genome U133Plus 2.0 arrays overnight. Hybridisation and scanning was performed in house at Barts Cancer Institute. For RT-qPCR analysis, RNA in the IP material was reverse-transcribed to cDNA using superscript III (Invitrogen) following the manufacturer’s instructions. Quantitative real time PCR was performed on ABI7500 equipment using gene-specific primer pairs and amplification condition of 2 min at 50°C, 10 min at 95°C, and then 40 cycles of 15 secs at 95°C and 45 secs at 60°C.

Total RNA was isolated using silica-based spin-column extraction kit (RNeasy mini kit, Qiagen) following the manufacturer’s protocol. Total RNA was treated with RNase-free DNase1 (Ambion) to reduce genomic DNA contamination. RNA integrity was evaluated using the Agilent Bioanalyzer. Two micrograms of total RNA was reverse transcribed with SuperScriptase III (Invitrogen) using Oligo-dT primers or random hexamers according to the manufacturer’s protocol. Negative (-RT) controls contained RNase-free water substituted for reverse transcriptase.

### Recombinant BORIS purification

The mammalian expression plasmid pM49-T4738 carries BORIS with an N-terminal HaloTag. Adherent HEK293T cells were transfected using Lipofectamine 2000 (Invitrogen) using standard methods. Cells were cultured for 48 h prior to harvest. Media were aspirated and cells washed in cold PBS before removal by cell scraping. Cells were centrifuged at 2000 × *g* for 5 min. The cell pellet containing over-expressed HaloTag-BORIS was stored at -80°C overnight. The cell pellet was lysed in lysis buffer (50 mM HEPES pH7.5, 150 mM NaCl) supplemented with BaculoGold protease inhibitor (BD Biosciences). HaloTag-BORIS was purified as per manufacturers protocol (Promega). The cell pellet was lysed on ice in 1 ml of lysis buffer per 2 × 10^7^ cells for 10 minutes, followed by 5 min pulse sonication (30 seconds ON, 30 seconds OFF) using Diagenode's Bioruptor 3 min (high setting at 4°C). Crude lysate was centrifuged at 10,000 × *g* for 30 min. The resulting cleared lysate was mixed with 100 ml HaloLink resin (25% slurry), incubated for 1 h rotating, and washed three times with lysis buffer. Washes were removed through centrifugation of the HaloLink resin at 1000 ×*g* for 5 min and aspiration. At the final wash, the resin was resuspended in cleavage buffer (lysis buffer supplemented with 15 mg/ml TEV protease) and rotated for 2 h at room temperature. Resin was centrifuged at 2000 x *g* for 5 min and supernatant removed. TEV protease was removed by the addition of HisLink resin to the supernatant and incubation for 20 min rotating at room temperature. HisLink was removed through centrifugation at 1000 × *g* for 5 min and the resulting supernatant snap frozen in liquid nitrogen and stored at -80°C. Quantification of the protein was carried out using BCA Protein Assay (Thermo Scientific). Purification was confirmed through Western blot analysis using rabbit anti-BORIS antibody (Abcam ab18337).

### Western blot analysis

Protein extracts or precipitated protein complexes were separated on a 4–12% gradient NuPAGE polyacrylamide gel (Invitrogen) and then blotted onto nitrocelluose membrane (Invitrogen) as described by Jones et al. [14]. After incubation with blocking solution (Tris-buffered saline containing 5% skimmed milk and 0.1% Tween-20) the membrane was incubated with corresponding antibodies overnight at 4°C. After several washes, bands were revealed with the corresponding horseradish peroxidase coupled secondary antibody and detected using the ECL detection kit (GE Healthcare) according to the manufacturer’s protocol. Densitometry scanning of the intensity of bands on the Western blot was quantified using ImageJ. The *p-values* were obtained using one-way ANNOVA test after intensity values were normalised to GAPDH levels.

### In vitro binding assay

For RNA and DNA binding assays, ~1 mg of purified BORIS protein was incubated with 125 nM of each biotinylated homopolymer in 400 ml of Binding Buffer (RBB, 150 mM NaCl, 20 mM Tris (pH7.5), 1 mM dithiothreitol [DTT] and 0.2% NP-40 at 4°C overnight. Nucleotide:protein complexes were isolated by addition of 20 ml prewashed Dynabeads M280 Streptavidin (Invitrogen) to the reaction for 30 min rotating at room temperature. Complexes were magnetically captured and washed three times in RBB. After the final wash, beads were resuspended in 10 ml NuPAGE LDS sample buffer supplemented with 5 mM DTT, heated to 70°C for 5 min. Captured proteins were resolved by 4 - 12% SDS/PAGE and analysed by Western blot using anti-BORIS antibody.

### Analysis of microarray data

Affymetrix Expression array files were analysed using Partek ® software, version 6.5 Copyright © 1993–2010 (Partek Inc.). Principle component analysis (PCA) was applied to identify any independent sources of variation in the data. We compared data for BORIS bound RNA transcripts with genome-wide gene expression profiles for each selected cell type (hNP1, 6dN and HEK293T cells) with at least two biological replicates. A t-test was performed and transcripts were considered to be preferentially associated with BORIS when the signals from the immunoprecipitated RNA fractions were enriched more than 2 fold, with a *p*-value < 0.01. The gene expression data have been deposited in NCBI’s Gene Expression Omnibus [27] and are accessible through GEO series accession number GSE42294 (http://www.ncbi.nlm.nih.gov/geo/query/acc.cgi?acc=GSE42294).

### Pathway analysis and functional classification

We used Protein ANalysis THrough Evolutionary Relationships (PANTHER) software to identify significantly enriched functional pathways and Gene Ontology (GO) terms associated with BORIS-bound transcripts [54]. Proteins were functionally classified using the PANTHER system (http://www.PANTHERdb.org).

### Quantitative real-time PCR

Both the published primers [16] and our own designed with Primer Express 2.0 were used in this study (Table 2). mRNA levels were quantified on an ABI7500 instrument using SYBR Green JumpStart Taq ReadyMix kit (Sigma-Aldrich) or platinium Taq polymerase kit (Invitrogen) with 50–100 ng of cDNA (except for BORIS primers when 150–200 ng of cDNA was used) and 100–200 nM primers. We used primers spanning the exon 4/5 junction of BORIS and findings were confirmed using published primers to exon 6/7 [15,16], and exon 9/10 [55] in a qRT-PCR assay with various concentrations of total cellular RNA. cDNA was generated using Oligo-dT or random primers approach. Use of 100 ng or less RNA resulted in inconsistent detection of BORIS. We therefore optimized our experiments using 150 ng total RNA for BORIS assays and 40 ng total RNA for the highly expressed CTCF and GAPDH assays. Absolute concentrations were estimated using standard curves generated from serial dilution of amplicons. The threshold cycle from serial dilutions of single stranded oligonucleotides was plotted against the log copy numbers of the target PCR products, and reported as copy numbers/μg of total RNA [56].

**Table 2:** List of primer pairs selected for RT-qPCR confirmation of BORIS associated transcripts in (A) hNP1 an hNP1 cells differentiated to neurons over 6 days (6dN), (B) HEK293T cells over-expressing BORIS

**A.**			
**Gene**	**Forward Primer**	**Reverse Primer**	**Exons**
RNF160	AAGTTTTGGAAGTATGGAAAACACA	GCTCACTTTGGATGCTTCCTCT	6-7
TTBK2	ATTGGCTGTGGGAGGAATGA	GTCTACCCAGCCGGAGAGTG	4-5
SKI	CTGGACGACGTGAAGGAGAAA	GGGACTGGGAAGAGGTGTCAT	1-2
PCLO	CACATGCACTGAGTGTCAAACC	CCGCCTAGAGCTCTTTTCATTT	3-4
APC	TGGAAGCAGAGAAAGTACTGGA	GATTCTGAAGTTGAGCGTAATACCA	4-5
BCL2	CCTGTGGATGACTGAGTACCTGA	TCTTCAGAGACAGCCAGGAGAA	1-2
HIP1	TGTAAAGGAAAAACACGCCAGA	TGTGGAACACATGGCAGAACTT	2-3
SOX4	GTTCGGCGTGTGCTTGG	GCAGCGCTTCCGTGTTCT	
APLP2	GCTCCTGCTTCTGCTGCTG	CCCATTTCCCAGTCTGAATGTT	1-2
CALR	CTCCCGATCCCAGTATCTATGC	TTGTTTCTCTGCTGCCTTTGTT	7-9
POU3F1	CAACAAGTGGCTGGAGGAGAC	GGCACTTGAGAAAGTGGCTCTC	
**B.**			
**Gene**	**Forward Primer**	**Reverse Primer**	**Exons**
CHD8	CATCGAGTGTTGGATAACTTCTCTG	ATCCATCATCATCAAGGGATCA	26-28
CTBP2	GAGAGTGATCGTGCGGATAGG	AGAGTCCGCTGTCTCTTCCAC	5-6
CTNNB1	TGAAAATCCAGCGTGGACAAT	GGTAAGACTGTTGCTGCCAGTG	2-3
FZD5	CGCTTCTCAGCGGAGTGAC	AGACGGTTAGGGCTCGGATT	1-2
LEF1	AATGAGAGCGAATGTCGTTGC	TCATAATATTTAGCCTGCTCTTCACG	7-8
LRP6	TTTATTGGGCAGATGCAAAACTTA	AATAACGTCAAGGCAAAAGGATGT	3-4
TLE1	TCCCCCTCACATGAGAGTACC	GAAAAGGGACAGGCTGCATCT	13-14
TCF4	ACCTCTTCCTGTACGCCTCCT	GATCTGGAGAATAGATCGAAGCAA	11-12
TCF3	TCTCGTCCAGCCCTTCTACC	CGTCCAGGTGGTCTTCTATCTTAC	13-15
WNT5A	CGACATCGAAGGTGGAACTG	CGTTCACCACCCCTGCT	3-4
APC	TGGAAGCAGAGAAAGTACTGGA	GATTCTGAAGTTGAGCGTAATACCA	4-5
TBP	CACGAACCACGGCACTGATT	TTTTCTTGCTGCCAGTCTGGAC	5-6

### Preparation and analysis of polysomes

Cell extracts for polysome analysis were prepared as described by Camacho-Vanegas O et al. [57]. Briefly, 5 x 10^8^ cells were incubated with cyclohexemide for 30 minutes then washed with ice-cold PBS containing 100 μg/ml cycloheximide (Sigma) to block ribosomes at the step of elongation. Cells were lysed for 5 minutes in cold 1 x polysome buffer (10 mM Tris–HCl pH 8.0, 140 mM NaCl, 1.5 mM MgCl_2_ and 0.05% NP40) containing 100 μg/ml cycloheximide. Cytoplasmic extracts were obtained after centrifugation at 10,000 × *g* for 5 min at 4°C, and then loaded onto a linear (10 – 50%) sucrose gradient in polysome buffer, and centrifuged at 100,000 × *g* for 2 h at 4°C. 650 μl fraction were collected and absorbance at 260 and 254 nm was measured using a spectrophotometer (Nanodrop). Aliquots of each fraction was mixed with 4 x PAGE loading buffer and analysed on a 4 – 12% NUPAGE gels.

### Cloning and transfection

The GFP-BORIS, GFP-CTCF and pEGFP-C3 vectors were transfected into HEK293T cells using FuGene 6-HD (Roche) according to manufacturer’s protocol as previously described [14].

### Activation of relative TCF/LEF-dual luciferase assay

The effect of BORIS on the WNT pathway was evaluated by measuring the activation of transcription factor TCF/LEF with the Cignal TCF/LEF reporter assay kit (SA Biosciences). In the first instance, HEK293T cells were cells co-transfected with TCF/LEF reporter constructs and either C3-BORIS or C3-empty vector, using Lipofectamin-2000 (Invitrogen) according to manufacturer's instructions. In other experiments, non-targeted or β-catenin siRNAs (Ambion) were combined with the C3 BORIS or C3 empty vector and co-transfected with TCF/LEF reporter constructs (SA Biosciences) according to manufacturer's instructions. The TCF/LEF reporter used a mixture of an inducible β-catenin-responsive luciferase construct and a constitutively expressing Renilla element (40:1). After 48 hrs incubation cells were collected and analyzed for TCF/LEF activity using a dual-luciferase assay kit (Promega-Biosciences). TCF/LEF activation values are expressed as arbitrary units using a Renilla reporter for internal normalization. Experiments were done in duplicate, and the standard deviations are indicated.

## Abbreviations

6dN: hNP1 cells differentiated over 6 days; BORIS (CTCFL): Brother of the Regulator of Imprinted Sites; CTCF: CCCTC-binding factor; hNP1: Human neural progenitor cells; RPL7: Ribosomal protein L7.

## Competing interests

The authors declare that they have no competing interests.

## Authors’ contributions

BWO and DS conceived the study and designed the experiments, BWO, TAJ, JA, JS, NO, DO, MAM, SP and CAP performed the experiments. TAJ performed and analysed Affymetrix array data, ARS provided reagents and advised on the manuscript, BWO, TAJ and DS wrote the paper. All authors read and approved the final manuscript.

## Supplementary Material

Additional file 1: Figure S1 Endogenous BORIS co-localises with newly synthesized RNA. HEK293T cells were pulse-labelled with 5’Fuorouridine (5’FU) for 10 minutes to label nascent RNA. Double immunoflorescence was performed with an anti-BrdU antibody to detect 5’FU (Red) and ant-BORIS (Green) as indicated. Nuclei were visualised with DAPI (blue). Click here for file

Additional file 2: Figure S2 Partial PCR amplification of endogenous BORIS in hNP1 cells and HEK293T cells. Gel electrophoresis analysis of fragments used for sequencing amplified with **(A)** primers +67F and +633R as previously described [22] or **(B)** with primers BORIS exon 9-10-11 forward: 5’-TGACCGCTCACATTCGTACC-3’ and reverse 5’-AGTGAACACGCAACCCGAAT-3’. Click here for file

Additional file 3: Table S1 Probes and transcripts associated with BORIS in hNP1 cells. Click here for file

Additional file 4: Table S2 Probes and transcripts associated with BORIS in hNP1 cells differentiated to neurons over 6 days (6dN). Click here for file

Additional file 5: Table S3 BORIS-associated transcripts up- or down-regulated during neural differentiation. Click here for file

Additional file 6: Figure S4 BORIS associates with RNA transcripts in stem cells and young neurons. BORIS associates with several transcripts (coloured blue) of the WNT signalling pathway. Click here for file

Additional file 7: Figure S5 Effects on protein levels after BORIS overexpression in HEK293T cells. Images and the associated densitometry measurements used to assess the protein levels of WNT5A/B and TCF3 after BORIS overexpression. Click here for file

Additional file 8: Figure S3 Confirmation of BORIS antibody specificity. Immuno-blotting of oligo-dT-RNA bound protein complexes from HEK293T cells transiently expressing CTCF (C3-CTCF), BORIS (C3-BORIS) or empty vector (C3-empty). Blot probed with anti BORIS antibodies. Click here for file

## References

[B1] Phillips JE Corces VG CTCF: Master Weaver of the Genome Cell 2009 137 7 1194 1211 10.1016/j.cell.2009.06.001 19563753 PMC3040116

[B2] Filippova GN Qi CF Ulmer JE Moore JM Ward MD Hu YJ Loukinov DI Pugacheva EM Klenova EM Grundy PE Tumor-associated zinc finger mutations in the CTCF transcription factor selectively alter tts DNA-binding specificity Cancer Res 2002 62 1 48 52 11782357

[B3] Ohlsson R Lobanenkov V Klenova E Does CTCF mediate between nuclear organization and gene expression? Bioessays 2010 32 1 37 50 10.1002/bies.200900118 20020479 PMC6375297

[B4] Filippova GN Fagerlie S Klenova EM Myers C Dehner Y Goodwin G Neiman PE Collins SJ Lobanenkov VV An exceptionally conserved transcriptional repressor, CTCF, employs different combinations of zinc fingers to bind diverged promoter sequences of avian and mammalian c-myc oncogenes Mol Cell Biol 1996 16 6 2802 2813 8649389 10.1128/mcb.16.6.2802 PMC231272

[B5] Zlatanova J Caiafa P CTCF and its protein partners: divide and rule? J Cell Sci 2009 122 Pt 9 1275 1284 19386894 10.1242/jcs.039990

[B6] Hore TA Deakin JE Marshall Graves JA The evolution of epigenetic regulators CTCF and BORIS/CTCFL in amniotes PLoS Genet 2008 4 8 e1000169 10.1371/journal.pgen.1000169 18769711 PMC2515639

[B7] Campbell AE Martinez SR Miranda JL Molecular architecture of CTCFL Biochem Biophys Res Commun 2010 396 3 648 650 10.1016/j.bbrc.2010.04.146 20438700

[B8] Klenova EM Morse HC 3rd Ohlsson R Lobanenkov VV The novel BORIS + CTCF gene family is uniquely involved in the epigenetics of normal biology and cancer Semin Cancer Biol 2002 12 5 399 414 10.1016/S1044-579X(02)00060-3 12191639

[B9] Hong JA Kang Y Abdullaev Z Flanagan PT Pack SD Fischette MR Adnani MT Loukinov DI Vatolin S Risinger JI Reciprocal binding of CTCF and BORIS to the NY-ESO-1 promoter coincides with derepression of this cancer-testis gene in lung cancer cells Cancer Res 2005 65 17 7763 7774 16140944 10.1158/0008-5472.CAN-05-0823

[B10] Ulaner GA Vu TH Li T Hu JF Yao XM Yang Y Gorlick R Meyers P Healey J Ladanyi M Loss of imprinting of IGF2 and H19 in osteosarcoma is accompanied by reciprocal methylation changes of a CTCF-binding site Hum Mol Genet 2003 12 5 535 549 10.1093/hmg/ddg034 12588801

[B11] Vatolin S Abdullaev Z Pack SD Flanagan PT Custer M Loukinov DI Pugacheva E Hong JA Morse H 3rd Schrump DS Conditional expression of the CTCF-paralogous transcriptional factor BORIS in normal cells results in demethylation and derepression of MAGE-A1 and reactivation of other cancer-testis genes Cancer Res 2005 65 17 7751 7762 16140943 10.1158/0008-5472.CAN-05-0858

[B12] Kang Y Hong JA Chen GA Nguyen DM Schrump DS Dynamic transcriptional regulatory complexes including BORIS, CTCF and Sp1 modulate NY-ESO-1 expression in lung cancer cells Oncogene 2007 26 30 4394 4403 10.1038/sj.onc.1210218 17260018

[B13] van de Nobelen S Rosa-Garrido M Leers J Heath H Soochit W Joosen L Jonkers I Demmers J van der Reijden M Torrano V CTCF regulates the local epigenetic state of ribosomal DNA repeats Epigenetics Chromatin 2010 3 1 19 10.1186/1756-8935-3-19 21059229 PMC2993708

[B14] Jones TA Ogunkolade BW Szary J Aarum J Mumin MA Patel S Pieri CA Sheer D Widespread Expression of BORIS/CTCFL in Normal and Cancer Cells PLoS One 2011 6 7 e22399 10.1371/journal.pone.0022399 21811597 PMC3139640

[B15] D'Arcy V Abdullaev ZK Pore N Docquier F Torrano V Chernukhin I Smart M Farrar D Metodiev M Fernandez N The potential of BORIS detected in the leukocytes of breast cancer patients as an early marker of tumorigenesis Clin Cancer Res 2006 12 20 Pt 1 5978 5986 17062669 10.1158/1078-0432.CCR-05-2731

[B16] D'Arcy V Pore N Docquier F Abdullaev ZK Chernukhin I Kita GX Rai S Smart M Farrar D Pack S BORIS, a paralogue of the transcription factor, CTCF, is aberrantly expressed in breast tumours Br J Cancer 2008 98 3 571 579 10.1038/sj.bjc.6604181 18195709 PMC2243163

[B17] Dougherty CJ Ichim TE Liu L Reznik G Min WP Ghochikyan A Agadjanyan MG Reznik BN Selective apoptosis of breast cancer cells by siRNA targeting of BORIS Biochem Biophys Res Commun 2008 370 1 109 112 10.1016/j.bbrc.2008.03.040 18355444

[B18] Hoffmann MJ Müller M Engers R Schulz WA Epigenetic control of CTCFL/BORIS and OCT4 expression in urogenital malignancies Biochem Pharmacol 2006 72 11 1577 1588 10.1016/j.bcp.2006.06.020 16854382

[B19] Kholmanskikh O Loriot A Brasseur F De Plaen E De Smet C Expression of BORIS in melanoma: lack of association with MAGE-A1 activation Int J Cancer 2008 122 4 777 784 10.1002/ijc.23140 17957795

[B20] Looijenga LH Hersmus R Gillis AJ Pfundt R Stoop HJ van Gurp RJ Veltman J Beverloo HB van Drunen E van Kessel AG Genomic and expression profiling of human spermatocytic seminomas: primary spermatocyte as tumorigenic precursor and DMRT1 as candidate chromosome 9 gene Cancer Res 2006 66 1 290 302 10.1158/0008-5472.CAN-05-2936 16397242

[B21] Risinger JI Chandramouli GV Maxwell GL Custer M Pack S Loukinov D Aprelikova O Litzi T Schrump DS Murphy SK Global expression analysis of cancer/testis genes in uterine cancers reveals a high incidence of BORIS expression Clin Cancer Res 2007 13 6 1713 1719 10.1158/1078-0432.CCR-05-2569 17363524

[B22] Woloszynska-Read A James SR Link PA Yu J Odunsi K Karpf AR DNA methylation-dependent regulation of BORIS/CTCFL expression in ovarian cancer Cancer Immun 2007 7 21 18095639 PMC2935752

[B23] Tiffen JC Bailey CG Marshall AD Metierre C Feng Y Wang Q Watson SL Holst J Rasko JE The cancer-testis antigen BORIS phenocopies the tumor suppressor CTCF in normal and neoplastic cells Int J Cancer 2013 133 7 1603 1613 10.1002/ijc.28184 23553099

[B24] Loukinov DI Pugacheva E Vatolin S Pack SD Moon H Chernukhin I Mannan P Larsson E Kanduri C Vostrov AA BORIS, a novel male germ-line-specific protein associated with epigenetic reprogramming events, shares the same 11-zinc-finger domain with CTCF, the insulator protein involved in reading imprinting marks in the soma Proc Natl Acad Sci U S A 2002 99 10 6806 6811 10.1073/pnas.092123699 12011441 PMC124484

[B25] Brown RS Zinc finger proteins: getting a grip on RNA Curr Opin Struct Biol 2005 15 1 94 98 10.1016/j.sbi.2005.01.006 15718139

[B26] Morrison AA Viney RL Ladomery MR The post-transcriptional roles of WT1, a multifunctional zinc-finger protein Biochim Biophys Acta 2008 1785 1 55 62 17980713 10.1016/j.bbcan.2007.10.002

[B27] Edgar R Domrachev M Lash AE Gene Expression Omnibus: NCBI gene expression and hybridization array data repository Nucleic Acids Res 2002 30 1 207 210 10.1093/nar/30.1.207 11752295 PMC99122

[B28] Fiorentino FP Macaluso M Miranda F Montanari M Russo A Bagella L Giordano A CTCF and BORIS Regulate Rb2/p130 Gene Transcription: A Novel Mechanism and a New Paradigm for Understanding the Biology of Lung Cancer Mol Cancer Res 2011 9 2 225 233 10.1158/1541-7786.MCR-10-0493 21325284

[B29] Renaud S Loukinov D Alberti L Vostrov A Kwon YW Bosman FT Lobanenkov V Benhattar J BORIS/CTCFL-mediated transcriptional regulation of the hTERT telomerase gene in testicular and ovarian tumor cells Nucleic Acids Res 2011 39 3 862 873 10.1093/nar/gkq827 20876690 PMC3035453

[B30] Nguyen P Cui H Bisht KS Sun L Patel K Lee RS Kugoh H Oshimura M Feinberg AP Gius D CTCFL/BORIS is a methylation-independent DNA-binding protein that preferentially binds to the paternal H19 differentially methylated region Cancer Res 2008 68 14 5546 5551 10.1158/0008-5472.CAN-08-1005 18632606 PMC2731476

[B31] Mi H Lazareva-Ulitsky B Loo R Kejariwal A Vandergriff J Rabkin S Guo N Muruganujan A Doremieux O Campbell MJ The PANTHER database of protein families, subfamilies, functions and pathways Nucleic Acids Res 2005 33 suppl 1 D284 D288 15608197 10.1093/nar/gki078 PMC540032

[B32] Klein PS Melton DA A molecular mechanism for the effect of lithium on development Proc Natl Acad Sci U S A 1996 93 16 8455 8459 10.1073/pnas.93.16.8455 8710892 PMC38692

[B33] Unsworth H Raguz S Edwards HJ Higgins CF Yague E mRNA escape from stress granule sequestration is dictated by localization to the endoplasmic reticulum FASEB J 2010 24 9 3370 3380 10.1096/fj.09-151142 20453113

[B34] Nolan RD Arnstein HR The dissociation of rabbit reticulocyte ribosomes into subparticles active in protein synthesis Eur J Biochem 1969 10 1 96 101 5345988 10.1111/j.1432-1033.1969.tb00660.x

[B35] Blobel G Isolation of a 5S RNA-protein complex from mammalian ribosomes Proc Natl Acad Sci U S A 1971 68 8 1881 1885 10.1073/pnas.68.8.1881 5001943 PMC389313

[B36] Cassiday LA Maher LJ III Having it both ways: transcription factors that bind DNA and RNA Nucleic Acids Res 2002 30 19 4118 4126 10.1093/nar/gkf512 12364590 PMC140532

[B37] Clemens KR Wolf V McBryant SJ Zhang P Liao X Wright PE Gottesfeld JM Molecular basis for specific recognition of both RNA and DNA by a zinc finger protein Science 1993 260 5107 530 533 10.1126/science.8475383 8475383

[B38] Hall TM Multiple modes of RNA recognition by zinc finger proteins Biochim Biophys Acta 2005 15 3 367 373 10.1016/j.sbi.2005.04.004 15963892

[B39] Ladomery M Sommerville J Binding of Y-box proteins to RNA: involvement of different protein domains Nucleic Acids Res 1994 22 25 5582 5589 10.1093/nar/22.25.5582 7530842 PMC310120

[B40] Scherrer T Femmer C Schiess R Aebersold R Gerber A Defining potentially conserved RNA regulons of homologous zinc-finger RNA-binding proteins Genome Biol 2011 12 1 R3 10.1186/gb-2011-12-1-r3 21232131 PMC3091301

[B41] Zhai G Iskandar M Barilla K Romaniuk PJ Characterization of RNA aptamer binding by the Wilms' tumor suppressor protein WT1† Biochemistry 2001 40 7 2032 2040 10.1021/bi001941r 11329270

[B42] Rosa-Garrido M Ceballos L Alonso-Lecue P Abraira C Delgado MD Gandarillas A A cell cycle role for the epigenetic factor CTCF-L/BORIS PLoS One 2012 7 6 e39371 10.1371/journal.pone.0039371 22724006 PMC3378572

[B43] Caricasole A Duarte A Larsson SH Hastie ND Little M Holmes G Todorov I Ward A RNA binding by the Wilms tumor suppressor zinc finger proteins Proc Natl Acad Sci U S A 1996 93 15 7562 7566 10.1073/pnas.93.15.7562 8755514 PMC38785

[B44] Font J Mackay JP Beyond DNA: zinc finger domains as RNA-binding modules Methods Mol Biol 2010 649 479 491 10.1007/978-1-60761-753-2_29 20680853

[B45] Mittal N Roy N Babu MM Janga SC Dissecting the expression dynamics of RNA-binding proteins in posttranscriptional regulatory networks Proc Natl Acad Sci U S A 2009 106 48 20300 20305 10.1073/pnas.0906940106 19918083 PMC2777960

[B46] Chenn A Walsh CA Regulation of cerebral cortical size by control of cell cycle exit in neural precursors Science 2002 297 5580 365 369 10.1126/science.1074192 12130776

[B47] Grigoryan T Wend P Klaus A Birchmeier W Deciphering the function of canonical Wnt signals in development and disease: conditional loss- and gain-of-function mutations of beta-catenin in mice Genes Dev 2008 22 17 2308 2341 10.1101/gad.1686208 18765787 PMC2749675

[B48] Zechner D Fujita Y Hulsken J Muller T Walther I Taketo MM Crenshaw EB 3rd Birchmeier W Birchmeier C: beta-Catenin signals regulate cell growth and the balance between progenitor cell expansion and differentiation in the nervous system Dev Biol 2003 258 2 406 418 10.1016/S0012-1606(03)00123-4 12798297

[B49] Greenbaum D Colangelo C Williams K Gerstein M Comparing protein abundance and mRNA expression levels on a genomic scale Genome Biol 2003 4 9 117 10.1186/gb-2003-4-9-117 12952525 PMC193646

[B50] Nie L Wu G Zhang W Correlation of mRNA expression and protein abundance affected by multiple sequence features related to translational efficiency in Desulfovibrio vulgaris: a quantitative analysis Genetics 2006 174 4 2229 2243 10.1534/genetics.106.065862 17028312 PMC1698625

[B51] Shin S Mitalipova M Noggle S Tibbitts D Venable A Rao R Stice SL Long-term proliferation of human embryonic stem cell-derived neuroepithelial cells using defined adherent culture conditions Stem Cells 2006 24 1 125 138 10.1634/stemcells.2004-0150 16100006

[B52] Kiesslich A von Mikecz A Hemmerich P Cell cycle-dependent association of PML bodies with sites of active transcription in nuclei of mammalian cells J Struct Biol 2002 140 1–3 167 179 12490165 10.1016/s1047-8477(02)00571-3

[B53] Keene JD Tenenbaum SA Eukaryotic mRNPs May represent posttranscriptional operons Mol Cell 2002 9 6 1161 1167 10.1016/S1097-2765(02)00559-2 12086614

[B54] Boyle EI Weng S Gollub J Jin H Botstein D Cherry JM Sherlock G GO::TermFinder—open source software for accessing Gene Ontology information and finding significantly enriched Gene Ontology terms associated with a list of genes Bioinformatics 2004 20 18 3710 3715 10.1093/bioinformatics/bth456 15297299 PMC3037731

[B55] Hines WC Bazarov AV Mukhopadhyay R Yaswen P BORIS (CTCFL) is not expressed in most human breast cell lines and high grade breast carcinomas PLoS One 2010 5 3 e9738 10.1371/journal.pone.0009738 20305816 PMC2840027

[B56] Bustin SA Absolute quantification of mRNA using real-time reverse transcription polymerase chain reaction assays J Mol Endocrinol 2000 25 2 169 193 10.1677/jme.0.0250169 11013345

[B57] Camacho-Vanegas O Weighardt F Ghigna C Amaldi F Riva S Biamonti G Growth-dependent and growth-independent translation of messengers for heterogeneous nuclear ribonucleoproteins Nucleic Acids Res 1997 25 19 3950 3954 10.1093/nar/25.19.3950 9380522 PMC146965

