# Supplementary figures and images for: BORIS/CTCFL is an RNA-binding protein that associates with polysomes
Source: BMC Cell Biol. 2013 Nov 26;14:52. doi: 10.1186/1471-2121-14-52 (PMC4219345; doi:10.1186/1471-2121-14-52)

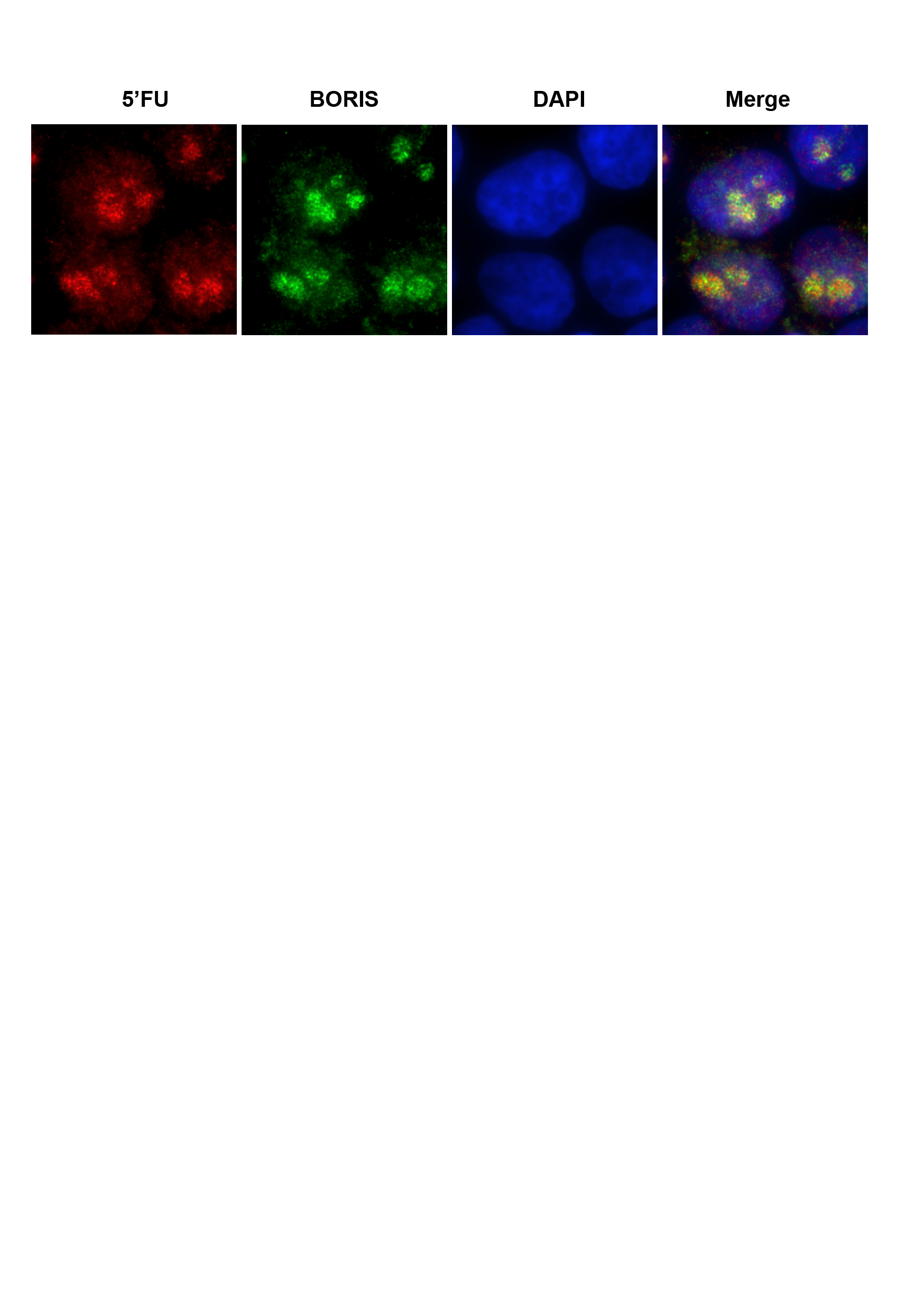

Supplement: Additional file 1: Figure S1 — Endogenous BORIS co-localises with newly synthesized RNA. HEK293T cells were pulse-labelled with 5’Fuorouridine (5’FU) for 10 minutes to label nascent RNA. Double immunoflorescence was performed with an anti-BrdU antibody to detect 5’FU (Red) and ant-BORIS (Green) as indicated. Nuclei were visualised with DAPI (blue). [file 1471-2121-14-52-S1.tiff]

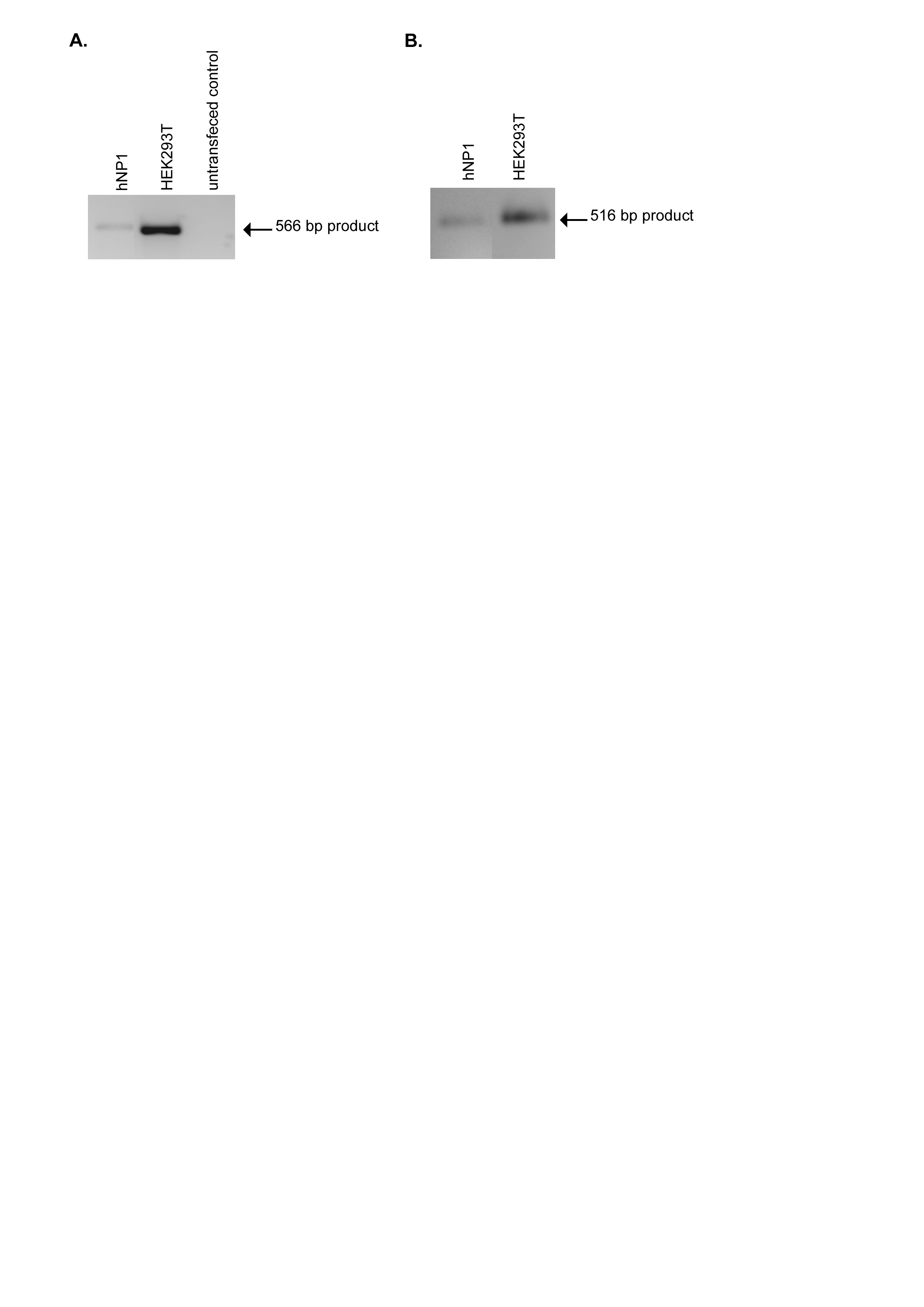

Supplement: Additional file 2: Figure S2 — Partial PCR amplification of endogenous BORIS in hNP1 cells and HEK293T cells. Gel electrophoresis analysis of fragments used for sequencing amplified with (A) primers +67F and +633R as previously described [22] or (B) with primers BORIS exon 9-10-11 forward: 5’-TGACCGCTCACATTCGTACC-3’ and reverse 5’-AGTGAACACGCAACCCGAAT-3’. [file 1471-2121-14-52-S2.tiff]

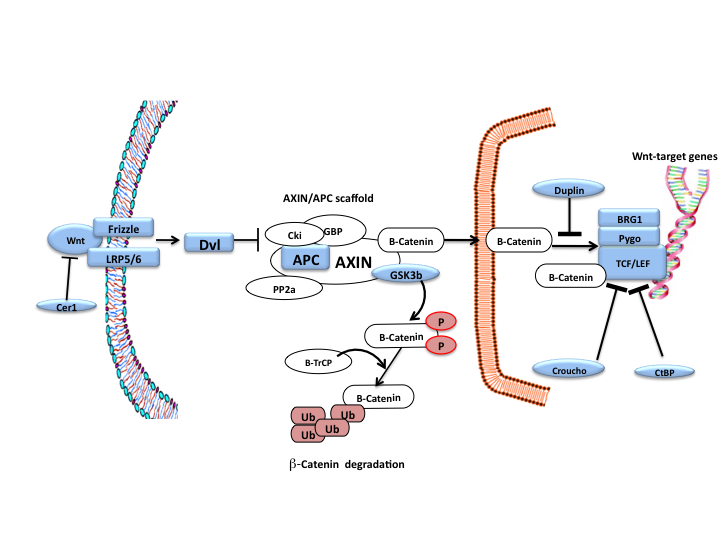

Supplement: Additional file 6: Figure S4 — BORIS associates with RNA transcripts in stem cells and young neurons. BORIS associates with several transcripts (coloured blue) of the WNT signalling pathway. [file 1471-2121-14-52-S6.tiff]

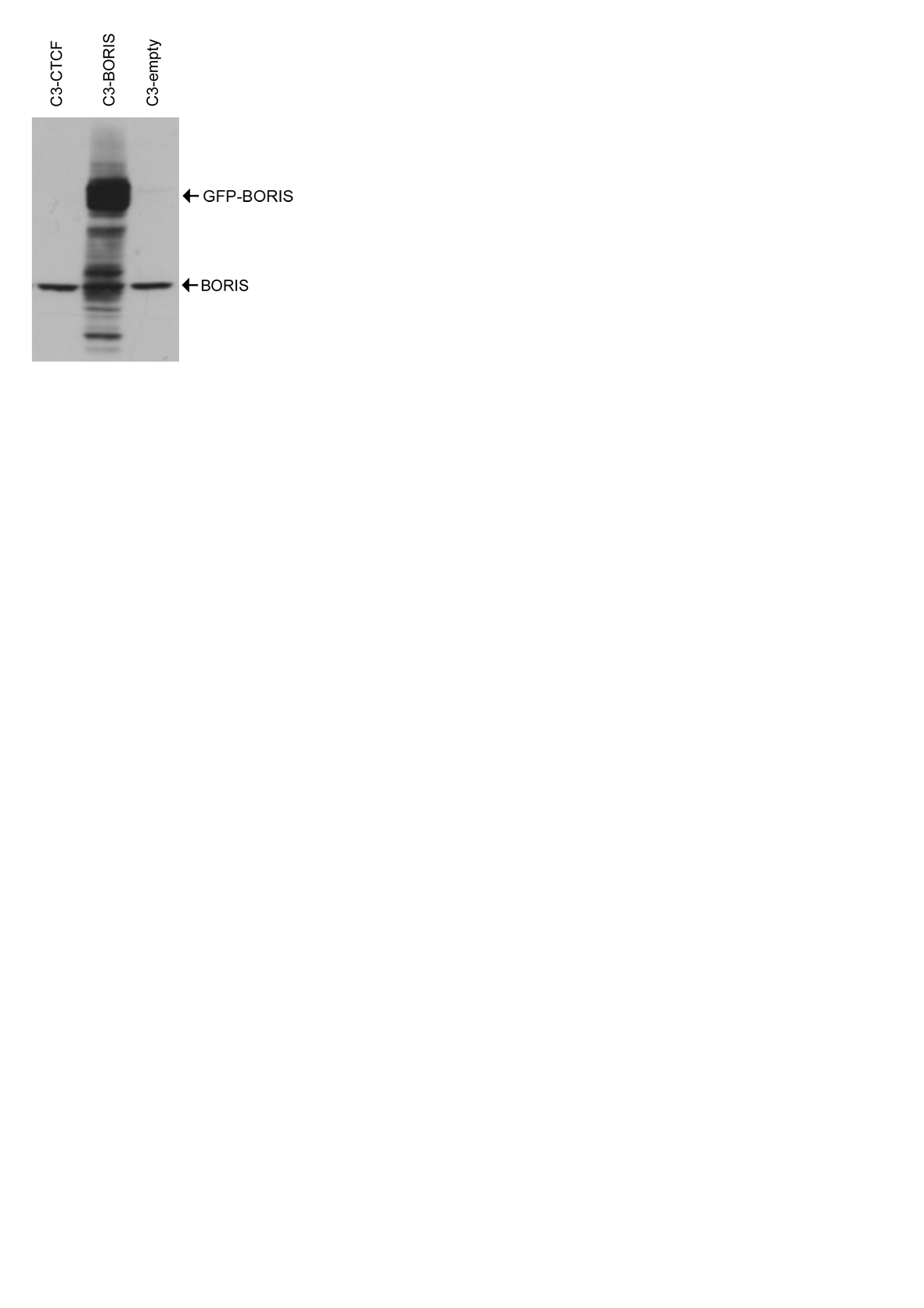

Supplement: Additional file 8: Figure S3 — Confirmation of BORIS antibody specificity. Immuno-blotting of oligo-dT-RNA bound protein complexes from HEK293T cells transiently expressing CTCF (C3-CTCF), BORIS (C3-BORIS) or empty vector (C3-empty). Blot probed with anti BORIS antibodies. [file 1471-2121-14-52-S8.tiff]
