# Supplementary material for: BORIS/CTCFL is an RNA-binding protein that associates with polysomes
Source: BMC Cell Biol. 2013 Nov 26;14:52. doi: 10.1186/1471-2121-14-52 (PMC4219345; doi:10.1186/1471-2121-14-52)
Supplement: Additional file 7: Figure S5 — Effects on protein levels after BORIS overexpression in HEK293T cells. Images and the associated densitometry measurements used to assess the protein levels of WNT5A/B and TCF3 after BORIS overexpression. [file 1471-2121-14-52-S7.pdf]

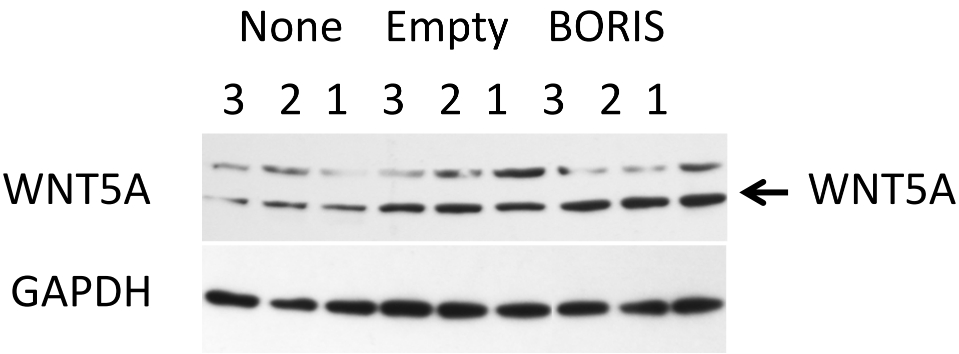

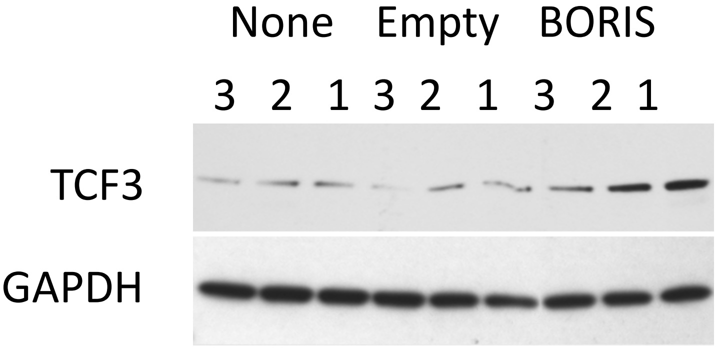

Measurements

	Wnt 5A	GAPDH (Wnt 5a)	GAPDH normalised
**Boris 1**	12630.27	17235.51	0.732805
**Boris 2**	11915.95	14799.803	0.805143
**Boris 3**	10956.42	15440.56	0.709587
**Empty 1**	8894.104	15629.368	0.569064
**Empty 2**	10249.05	16360.489	0.626452
**Empty 3**	9131.861	17650.489	0.517372
**None 1**	6000.083	14591.368	0.411208
**None 2**	7000.447	10497.004	0.6669
**None 3**	4350.669	14756.853	0.294824

	TCF3	GAPDH (TCF3)	GAPDH normalised
**Boris 1**	10983.18	14835.439	0.740334
**Boris 2**	8180.276	13925.439	0.587434
**Boris 3**	4703.083	13789.853	0.341054
**Empty 1**	3307.255	11428.903	0.289376
**Empty 2**	2901.548	15010.439	0.193302
**Empty 3**	854.527	16381.731	0.052163
**None 1**	3105.719	16466.853	0.188604
**None 2**	3486.669	17419.631	0.200157
**None 3**	2742.669	16200.338	0.169297
